# Risk Factors for Motor Vehicle Collisions in Patients with Primary Open-Angle Glaucoma: A Multicenter Prospective Cohort Study

**DOI:** 10.1371/journal.pone.0166943

**Published:** 2016-11-29

**Authors:** Kenya Yuki, Sachiko Awano-Tanabe, Takeshi Ono, Daisuke Shiba, Hiroshi Murata, Ryo Asaoka, Kazuo Tsubota

**Affiliations:** 1 Department of Ophthalmology, Keio University School of Medicine, Tokyo, Japan; 2 Department of Ophthalmology, the University of Tokyo, Graduate School of Medicine, Tokyo, Japan; Massachusetts Eye & Ear Infirmary, Harvard Medical School, UNITED STATES

## Abstract

**Purpose:**

To identify the incidence rate of motor vehicle collisions (MVCs) in patients with no ocular pathology other than primary open-angle glaucoma (POAG) and determine the putative risk factors for MVCs in this group of patients.

**Methods:**

We designed a prospective cohort study across three centers utilizing a consecutive sampling method to identify all patients with POAG between the ages of 40 and 80 years old. Patients with glaucoma were consecutively screened for eligibility. All study participants answered a questionnaire about motor vehicle collisions at baseline, and answered the questionnaire again every 12 months (± 1 month) after baseline for three years. A binocular integrated visual field was calculated for each patient by merging a patient’s monocular Humphrey Field Analyzer (HFA) visual fields (VFs), using the ‘best sensitivity’ method. Patients with incident MVCs were defined as the “MVC+” group and patients without incident MVCs were defined as the “MVC-" group. Adjusted odds ratios for the incidence of MVCs were estimated with a logistic regression model.

**Results:**

One hundred and ninety-one Japanese POAG patients were analyzed in this study. The age of the participants was 63.7 ± 10.2 [mean ± standard deviation]. A total of 28 participants experienced a MVC during the follow up period of three years (4.9% per year). Ten patients (5.2%) experienced a MVC in the first year, 13 patients (6.8%) in the second year, and 11 patients (5.8%) in the third year (some patients experienced multiple MVCs over different years). Best corrected visual acuity in the worst eye was significantly worse in the MVC+ group (0.03 ± 0.01, mean ± standard deviation, LogMar) compared with the MVC- group (0.01 ± 0.003, p = 0.01), and was the only variable identified as a significant predictor of future MVCs in the multiple logistic regression model [odds ratio: 1.2, 95% confidence interval (CI): 1.1 to 1.4].

**Conclusion:**

Deterioration in visual acuity in the worst eye is a risk factor for future MVCs in patients with POAG.

## Introduction

Motor vehicle collision (MVC) is a serious public health concern worldwide. More than 30,000 people were killed in MVCs in the USA in 2013 alone.[1] In 2012, 3,393 patients suffered spinal cord injuries in the USA and about 30% were the direct result of an MVC.[2] It is estimated that deaths and injuries resulting from MVCs cost the global community about 518 billion US dollars every year.[3]

Glaucoma is a disease characterized by progressive retinal ganglion cell loss. Glaucoma usually manifests as peripheral visual field (VF) loss which may progress to central VF damage, and visual acuity impairment. Glaucoma is the second leading cause of blindness in the world affecting more than 60 million people.[4] Aging is a significant risk factor for glaucoma.[5] Thus, as the number of elderly drivers continues to grow, both in developed and developing countries, so will the prevalence of drivers with glaucoma.

Healthy vision is clearly one of the most important attributes required to drive safely. Several studies have evaluated the association between glaucoma and MVCs.[6–13] We previously reported that patients with severe glaucoma (n = 20, prevalence of MVCs was 25.0%) were 8 times more likely to be involved in MVCs compared with controls (n = 144, prevalence of MVCs was 3.5%).[14] Recently Kwon et al. reported that the rate of MVCs was 1.65 times higher in drivers with glaucoma (n = 206, 37 subjects (18%) had a history of MVCs) compared with those without glaucoma (n = 1,794, 219 subjects (13%) had a history of MVCs).[8] These results suggest that glaucoma is significantly associated with an increased likelihood of MVC occurrence. However, these studies were conducted in a cross sectional manner so it is possible that persons with glaucoma may simply recall a higher number of MVCs. It is of interest to investigate the relationship between visual function and MVC incidence in a prospective manner. The aim of the present study is to survey the incidence of MVCs in patients with primary open angle glaucoma (POAG) and investigate risk factors for future MVCs.

## Subjects and Methods

This study's procedures conformed to the tenets of the Declaration of Helsinki and to national (Japanese) and institutional (Keio University School of Medicine) regulations. The study was approved by the Ethics Committee of the Keio University School of Medicine (#2010293). All study participants gave informed, written consent prior to being enrolled.

### Study Design and Patient Enrolment

This was a prospective cohort study. Japanese patients between 40 and 85 years of age who visited Keio University Hospital (Tokyo, Japan), the Iidabashi Eye Clinic (Tokyo, Japan), or the Tanabe Eye Clinic (Yamanashi, Japan) between May 1, 2011 and November 30, 2011 were screened for eligibility.

### Baseline Evaluation of Patients with Glaucoma

Patients with glaucoma were consecutively screened for eligibility using a battery of ophthalmic examinations, including slit-lamp biomicroscopy, funduscopy, gonioscopy, intraocular pressure measurements by Goldmann applanation tonometry, and VF examination with a Humphrey visual field analyser (HFA) and the 24–2 Swedish Interactive Threshold Algorithm Standard Strategy (Carl Zeiss Meditec, Dublin, CA). The findings were analysed by T.S., and K.Y., both of whom subspecialize in glaucoma. The reliability of VF measurements was confirmed to be high with tests required to have less than a 20% fixation loss rate and less than a 15% false-positive rate.[15]

### Diagnostic Criteria for POAG

POAG was diagnosed when three findings were present: (1) glaucomatous optic cupping, represented by notch formation, generalized cup enlargement, a senile sclerotic or myopic disc, or nerve-fibre layer defects; (2) glaucomatous VF defects, defined according to Anderson and Patella’s criteria (a cluster of 3 or more points in the pattern deviation plot within a single hemifield [superior or inferior] with a p value < 5%, one of which must have a p value <1%) [16]; and (3) an open angle observed on gonioscopy.

### Exclusion Criteria

Participants were excluded if they had an ophthalmologic disease other than POAG that could compromise visual acuity or contribute to VF loss. Participants were also excluded if they had a decimal best corrected visual acuity (BCVA) of less than 0.7, if they did not have a driver's license or drove 1 kilometer or less per week, or had a mental disorder that prevented them from understanding the questionnaire.

### Baseline Questionnaire of Motor Vehicle Collisions and Driving Behaviour

All study participants answered the following questionnaire in Japanese (translated here) at baseline ophthalmic examination:

1. Do you have a driver's license? (Yes/No/Previously)
2. How long have you driven/did you drive a car? (__years)
3. How many kilometres per week do you normally drive? (__km)
4. Have you been involved in one or more traffic accidents in the past five years, including a single-car or minor accident, in which you were driving the car? (Yes/No)
5. How many traffic accidents have you ever been involved in, in the past five years? (__)

Demographic information recorded included age, sex, height, weight, alcohol intake (yes/no), smoking history (yes/no/previous), current and previous illnesses (e.g., systemic hypertension, diabetes mellitus, depression, brain infarction), and medical history, including oral medications such as sleeping aids, anti-hypertensive drugs, or tranquilizers.

### Follow-up Questionnaire of Motor Vehicle Collisions

All study participants answered the following questionnaire in Japanese (translated here) every 12 months (± 1 month) after the baseline questionnaire:

1. Do you have a driver's license? (Yes/No/Previously)
2. How many kilometres per week do you normally drive? (__km)
3. Have you been involved in one or more traffic accidents in the past one year, including a single-car or minor accident, in which you were driving the car? (Yes/No)

Patients who answered ‘Yes’ to question number 3 were defined as having experienced a MVC.

### Integrated Binocular Visual Field

A binocular integrated visual field (IVF) was calculated for each patient by merging a patient’s monocular HFA VFs, using the ‘best sensitivity’ method, where the IVF total deviation (TD) at each point was calculated using the maximum TD (least negative) value from each of the two overlapping points, as if the person was viewing the field binocularly. [17] The IVF mean deviation (MD) was calculated as the mean of 52 TD values across the VF. We were unable to obtain IVF data for 8 POAG patients, and these were excluded from the following analyses.

### Statistical Analysis

Descriptive statistics were calculated for the demographic, medical, and visual-function variables for patients with a history of MVCs and patients without a history of MVCs, and compared using the unpaired t-test, the Mann-Whitney U test, or the Chi-squared test. Patients with incident MVCs were defined as the “MVC+” group and patients without incident MVCs were defined as the “MVC-" group.

Adjusted odds ratio and 95% confidence intervals for the incidence of MVCs were estimated with the multiple logistic regression model to examine the effects of the following (possible confounding) factors on unadjusted results, using the forced-entry method: age, sex, best corrected visual acuity in the worst eye, best corrected visual acuity in the better eye, MD value in the worst eye, body mass index, driving distance per week, and history of MVCs in the past five years. These parameters were selected as independent variables because they were deemed clinically important risk factors.

A p-value less than 0.05 was considered statistically significant. Decimal visual acuity was converted to LogMAR visual acuity for analysis. All analyses were performed using the statistical programming language ‘R’ (R version 2.15.1; The Foundation for Statistical Computing, Vienna, Austria)

## Results

Of the 431 consecutive POAG patients screened, 204 were excluded. The reasons for excluding participants were as follows (the numbers in parentheses indicate the number of persons excluded): refusal to participate (3), dementia (2), post retinal-detachment (20), diabetic retinopathy (21), bullous keratopathy (2), age-related macular degeneration (5), other ocular disease (7), never had a driver’s license (73), had already returned driver's license (15), and drive less than 1 kilometer per week (56). Consequently 227 POAG patients were included in this prospective study. Among 227 POAG patients who answered the baseline questionnaire, 191 POAG patients answered the follow-up MVC questionnaires over a period of three years. The proportion of patients lost to follow-up was 15.9% (36/227). The average age of patients was 63.7 ± 10.2 [average ± standard deviation (S.D.)]. 141 participants were male and 50 were female. Among the 191 patients, 28 experienced a MVC over the three year follow-up (4.9% per year); 10 (5.2%), 13 (6.8%) and 11 (5.8%) patients experienced MVCs in the first, second and third year, respectively. One patient experienced a MVC in both the first and second years, 3 participants experienced a MVC in both the second and third years, and 1 person experienced a MVC in all three years.

The comparison of systemic and ocular measurements, including BCVA in the better eye and in the worst eye, MD in the better eye and in the worst eye, is shown in Table 1. BCVA in the worst eye was significantly worse in the MVC+ group compared with that in the MVC- group. There was no significant difference in BCVA in the better eye, MD in the better eye, MD in the worst eye, and IVF-MD between the two groups. We evaluated risk factors associated with future MVCs using the multiple logistic regression model with incident MVCs as the dependent variable and age, sex, BMI, BCVA in the worst eye, BCVA in the better eye, IVF-MD, distance driven per week, and past history of MVCs as independent variables. The results are shown in Table 2. BCVA in the worst eye was found to be a significant predictor of future MVCs in this analysis.

**Table 1:** Comparison of various visual and systemic parameters between MVC+ group and MVC- group.

	MVC+ (mean ± s.d.)	MVC- (mean ± s.d.)	P value
N	28	163	
Age (years)	66.9 ± 11.4	63.1 ± 9.6	0.026
Sex (male/female)	20/8 (71.4%/28.6%)	121/43 (74.2%/25.8%)	0.76
B.M.I	22.4 ± 2.5	22.7 ± 3.0	0.84
BCVA in the better eye (LogMar)	0.00 ±0.00	0.00 ± 0.02	0.21
BCVA in the worst eye (LogMar)	0.03 ± 0.01	0.01 ± 0.003	0.01
MD in the better eye (dB)	-3.0 ± 4.0	-2.5 ± 4.0	0.35
MD in the worst eye (dB)	-7.1 ± 7.2	-7.0 ± 6.2	0.71
IVF-MD (dB)	-2.1 ± 3.9	-1.6 ± 3.7	0.55
Glaucoma severity#			0.52
Mild	18 (64.3%)	88 (54.0%)	
Moderate	4 (14.3%)	42 (25.8%)	
Severe	6 (22.4%)	33 (20.2%)	
Past history of MVCs (Yes/No)	9/28 (32.1%)	21.5% = 35/163	0.23
Driving distance per week (km)	107.9 ± 180.5	98.3 ± 89.2	0.12
Prevalence of hypertension	7/28 (25.0%)	57/163 (35.0%)	0.30
Prevalence of diabetes mellitus	4/28 (14.3%)	28/163 (17.2%)	0.71
Smoking status			0.34
Never	18 (64.3%)	82 (50.3%)	
Current	2 (7.1%)	27 (16.6%)	
Previous	8 (28.6%)	54 (33.1%)	
Alcohol intake			0.65
Never	12 (42.9%)	76 (46.6%)	
Sometimes	6 (21.4%)	43 (26.4%)	
Daily	10 (35.7%)	44 (27.0%)	

#Glaucoma severity was defined by MD in the worst eye using Mills definition

Abbreviation: MVCs, motor vehicle collisions; B.M.I, body mass index; BCV A, best corrected visual acuity; Logmar, logarithm of the Minimum Angle of Resolution; MD, mean deviation; dB, decibel; IVF, integrated visual field.

**Table 2:** The odds ratio for motor vehicle collision occurrence in patients with POAG.

Variable	odds ratio	95% CI	P
Age (years)	1.0	[0.99 to 1.1]	0.08
BCVA in the worst eye. LogMar. (for increase of 0.01)	1.2	[1.1 to 1.4]	0.003
BCVA in the better eye. LogMar. (for increase of 0.001)	0.68	[0.0 to 221]	0.99
BMI (kg/m^2^)	0.9	[0.8 to 1.1]	0.22
Gender (male as reference)	1.0	[0.4 to 2.7]	0.37
IVF-MD (dB) (for increase of 1 dB)	0.95	[0.8 to 1.1]	0.37
History of MVC	0.92	[0.86 to 6.2]	0.09
Driving distance per week (km)	1.0	[0.996 to 1.0]	0.59

## Discussion

In this prospective study we found that visual acuity in the worst eye is a significant risk factor for MVCs in patients with POAG. Interestingly, this result contradicts findings from many previous studies. Kwon et al. reported that worst eye visual acuity in people with glaucoma was not associated with MVCs in a population-based study.[7] Gracitelli et al. also reported that visual acuity both in the better and worst eye were not predictors of MVCs in patients with glaucoma in a prospective study.[18] Further, two recent well-designed cohort studies did not find a significant relationship between visual acuity and MVC rates in healthy people.[19,20] Good visual function is undoubtedly important for safe driving and we have previously reported that poor visual acuity in the better eye is associated with MVCs in patients with POAG in a cross-sectional study.[11]

The reason for the discrepancy between our current results and findings from previous studies is unclear. However, there are important design differences between the present study and the previous research that may influence findings. Three of the previous studies evaluated the association between binocular visual acuity and MVCs, and reported no significant association.[7,19,20] Binocular visual acuity is similar to BCVA in the better eye so these papers are not directly comparable with our study. The research by Gracitelli et al. on the other hand did compare better eye BCVA and worst eye BCVA between MVC+ and MVC- groups; however, the authors reported no significant difference.[18] There are a number of possible explanations for this; firstly, the average BCVA in their MVC+ group was 0.12 (LogMar) while in the MVC- group it was 0.06 (LogMar). Although, there was no significant statistical difference, the MVC+ group had worse BCVA than the MVC- group. Indeed this difference is larger than observed in our study. Therefore, it is possible that the Gracitelli et al. study was underpowered to detect a statistically significant difference, because the size of their MVC+ group was only 11. Furthermore, Owsley et al. have speculated that people with worse binocular visual acuity tend to drive less, so any excess MVC risk they pose could be diminished.[21] Conversely, people with worse visual acuity in the worst eye may not be aware that they have a visual acuity impairment, which would increase their risk of MVCs. It is also important to note that most patients in the current study were in a relatively early stage of glaucoma. Visual acuity is usually preserved until very late stage glaucoma [22], and this could be the reason why visual acuity in the worst eye, not the better eye, was a risk factor for MVCs. It would be of interest to survey MVCs in a population with moderate to advanced stage glaucoma; in these patients there may be a significant relationship between visual field indices and MVCs.

In the current study, no significant association between VF defects and the risk of future MVCs was found. The association between glaucomatous VF defects and MVCs is debatable.[6,7,9–11,15,23] Kwon et al.–in a population-based retrospective study–reported that drivers with glaucoma had a 1.7 times higher rate of MVCs compared with those without glaucoma.[7] They also reported that glaucomatous defects in the left, upper and lower VF regions were associated with a higher MVC rate compared with defects in the right, horizontal, or vertical regions. Huisingh et al. reported that drivers with severe binocular VF impairment in the overall field were 40% more likely to be at-fault for MVCs.[24] However, Gracitelli et al.–in a prospective study of patients with glaucoma–reported that binocular VF mean sensitivity, better eye MD and worst eye MD in standard automated perimetry (SITA standard 24–2) were not associated with increased risk of MVCs.[18] In a previous cross-sectional study, we also failed to show an association between binocular central VF sensitivity (as measured by SITA standard 24–2 VFs) and MVCs.[12] Like us, Kwon et al. measured patients’ VFs using the HFA, but they measured the widest possible horizontal extent for the field that could be tested for each eye (< 60°). Huisingh et al. also carried out visual field testing with a horizontal extent of < 60°. These results may suggest that limited damage to the binocular central visual field is not associated with MVCs, but damage to the peripheral visual field (wider than 30 degrees) could be associated with MVCs in patients with glaucoma.

Szlyk et al. investigated the driving performance of people with glaucoma and found that performance correlated with three Goldmann visual field measures: combined horizontal extent, total horizontal extent and total peripheral extent; however, performance was not correlated with visual field parameters measured by Humphery visual field testing within 24 degrees.[25] Thus, this report also supports our hypothesis that peripheral visual field loss is associated with driving ability, and, therefore with MVCs.

In previous studies, we have reported that damage to the visual field in the worst eye is associated with MVCs.[13,14] The results from the current study are inconsistent with our previous report. The reason for this is unclear but one possible explanation is the difference in study design. In this current study, all participants were glaucoma patients with a visual field defect but in the previous study control subjects were included with no visual field defect. Another possible explanation is that here we conducted a prospective study with a recall period of one year while previous studies were cross-sectional with a recall period of 5 to 10 years. The results of the cross sectional studies may therefore be influenced by longer recall periods.

**Fig 1:**
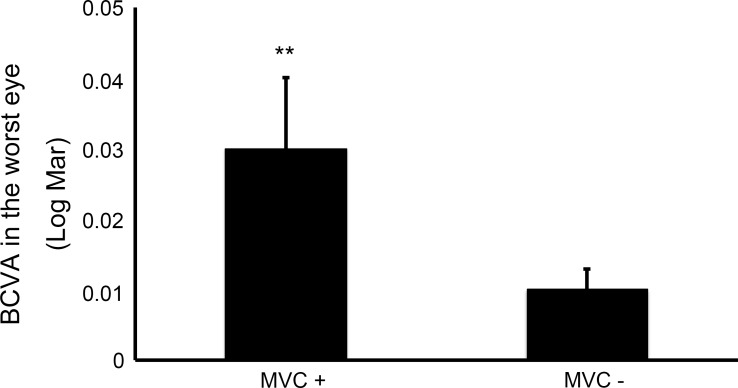
BCVA in the worst eye was significantly worse in the MVC+ group than that in the MVC- group (p = 0.01). Abbreviations: BCVA, best corrected visual acuity, MVC, motor vehicle collision, Log Mar, logarithms of minimum angle of resolution.

The difference in visual acuity in the worst eye found in this study is very subtle, with mean BCVA (logMAR) 0.03 ± 0.01 in the MVC+ group and 0.01 ± 0.003 in the MVC- group (Fig 1). Furthermore, as VF defects were not associated with MVC in this study, poor visual acuity in the worst eye could indicate the involvement of other diseases apart from POAG. However, every effort was made to exclude patients with other disorders that could affect their vision, including diabetic retinopathy, age-related macular degeneration, epi-retinal membrane, and severe cataract. Thus, the cause of visual acuity loss should be largely attributed to POAG or mild cataract.

### Limitations

A significant limitation of our study is that we relied on self-reporting of MVCs as the main outcome. There may be a reluctance, from some patients, to honestly provide this information. However, reliance on police reports would mean that some MVCs, not meeting the legal criteria for having to report a MVC, would be missed. Self-reported and police/state records provide complementary information for the ascertainment of MVCs, but self-reported records have yielded more events than state records in previous reports.[6, 26] A second limitation of the study is that a control arm was not included; therefore, we could not compare the incidence of MVCs in control subjects against glaucoma patients. Third, we did not measure binocular visual acuity. Binocular visual acuity is an important indicator, because in real life, we generally use binocular vision. We intend to analyze the importance of binocular visual acuity in a future study. Finally, we were unable to follow all the participants over the three year period; participants who were ‘lost to follow-up’ could introduce a bias in our results if the reasons for leaving the study were associated with the study outcome.

## Conclusion

Poor visual acuity in the worst eye is a risk factor for future MVCs in patients with POAG in Japan. Maintaining visual acuity in POAG patients may reduce MVC occurrence.

## Supporting Information

S1 File Data analyzed (CSV)

## Abbreviations

CI: confidence interval
POAG: primary open-angle glaucoma
BCVA: best corrected visual acuity
LogMar: log of a minimum angle of resolution
BMI: body mass index
IVF-MD: integrated visual field—mean deviation
dB: decibel
MVC: motor vehicle collision
